# Predicting Treatment Outcomes from Prefrontal Cortex Activation for Self-Harming Patients with Borderline Personality Disorder: A Preliminary Study

**DOI:** 10.3389/fnhum.2016.00220

**Published:** 2016-05-18

**Authors:** Anthony C. Ruocco, Achala H. Rodrigo, Shelley F. McMain, Elizabeth Page-Gould, Hasan Ayaz, Paul S. Links

**Affiliations:** ^1^Department of Psychology, University of Toronto ScarboroughToronto, ON, Canada; ^2^Centre for Addiction and Mental Health, Borderline Personality Disorder ClinicToronto, ON, Canada; ^3^Department of Psychology, University of TorontoToronto, ON, Canada; ^4^School of Biomedical Engineering, Science and Health Systems, Drexel UniversityPhiladelphia, PA, USA; ^5^Department of Family and Community Health, University of PennsylvaniaPhiladelphia, PA, USA; ^6^Division of General Pediatrics, Children’s Hospital of PhiladelphiaPhiladelphia, PA, USA; ^7^Department of Psychiatry, Western UniversityLondon, ON, Canada

**Keywords:** borderline personality disorder, self-harm, dialectical behavior therapy, prefrontal cortex, impulse control, fNIRS

## Abstract

Self-harm is a potentially lethal symptom of borderline personality disorder (BPD) that often improves with dialectical behavior therapy (DBT). While DBT is effective for reducing self-harm in many patients with BPD, a small but significant number of patients either does not improve in treatment or ends treatment prematurely. Accordingly, it is crucial to identify factors that may prospectively predict which patients are most likely to benefit from and remain in treatment. In the present preliminary study, 29 actively self-harming patients with BPD completed brain-imaging procedures probing activation of the prefrontal cortex (PFC) during impulse control prior to beginning DBT and after 7 months of treatment. Patients that reduced their frequency of self-harm the most over treatment displayed lower levels of neural activation in the bilateral dorsolateral prefrontal cortex (DLPFC) prior to beginning treatment, and they showed the greatest increases in activity within this region after 7 months of treatment. Prior to starting DBT, treatment non-completers demonstrated greater activation than treatment-completers in the medial PFC and right inferior frontal gyrus. Reductions in self-harm over the treatment period were associated with increases in activity in right DLPFC even after accounting for improvements in depression, mania, and BPD symptom severity. These findings suggest that pre-treatment patterns of activation in the PFC underlying impulse control may be prospectively associated with improvements in self-harm and treatment attrition for patients with BPD treated with DBT.

## Introduction

Borderline personality disorder (BPD) is a mental disorder characterized by difficulties with emotion regulation, impulse control, self-image and interpersonal relationships (American Psychiatric Association, 2013). Deliberate self-injury with or without the intent to die, commonly referred to as *self-harm*, occurs in 63–80% of patients with BPD (Chapman et al., 2005). A treatment that reduces self-harm in patients with BPD is dialectical behavior therapy (DBT), an empirically-supported psychotherapy intended to improve behavioral control and emotion regulation (Linehan et al., 2006; McMain et al., 2009). Whereas DBT is an effective treatment, outcomes in clinical trials represent averages and the effect on any one individual may be larger or smaller than the average effect. Response heterogeneity may be explained in part by meta-analytic evidence that suggests patients who begin but do not complete treatment have worse outcomes over time (McMurran et al., 2010). Additionally, more than one-quarter of patients with BPD in DBT end treatment prematurely (Linehan et al., 2006; McMain et al., 2009). Therefore, determining factors that predict treatment response and attrition will be useful for advancing our understanding of how to adapt treatment to address the needs of individuals.

Neuroimaging holds promise for isolating markers of brain function that predict treatment outcomes for self-harming patients with BPD. In major depression, psychotherapy studies examining neural activation while viewing negative emotional pictures have found that activity in amygdala, anterior cingulate, and dorsolateral prefrontal cortex (DLPFC), may be prospectively associated with responses to cognitive-behavioral treatments (for a review, see DeRubeis et al., 2008). Patients with BPD show neural systems dysfunctions in similar regions while processing negative emotions (Ruocco et al., 2013) and two small preliminary studies suggest that activity in these same regions may be modulated by DBT and potentially associated with BPD symptom improvements (Schnell and Herpertz, 2007; Goodman et al., 2014). To our knowledge, no studies have yet examined neural activation during response inhibition as a potential predictor of psychotherapy outcomes for any mental disorder. Deficits in response inhibition may underlie BPD and self-harm (Ruocco, 2005; Ruocco et al., 2012; Williams et al., 2015) and considering that DBT is intended to improve behavioral control and reduce self-harm in BPD, activation in neural regions underlying response inhibition, specifically motor inhibitory control, may conceivably show associations with treatment outcomes.

The current preliminary study probed activation in the PFC associated with motor inhibitory control before patients enrolled in a standard outpatient DBT program and again after 7 months of treatment. The primary aims of the study were to determine whether pre-treatment activation in the PFC was associated with: (1) reductions in self-harm through treatment; and (2) attrition from treatment. We hypothesized that patients with BPD who go on to demonstrate the greatest reductions in self-harm would show lower pre-treatment activity in lateral regions of the PFC underlying impulse control, consistent with the notion that these patients may have the most to gain from a treatment intended to improve behavioral control. Furthermore, we anticipated that patients who initiate treatment but do not complete treatment would display higher levels of activity in these same regions of the PFC, which could represent a neurophysiological marker signifying increased risk for treatment attrition in DBT. Exploratory analyses investigated associations between shifts in PFC activation and changes in self-harm between pre-treatment and seven-month assessments.

## Materials and Methods

### Participant Characteristics

**Figure 1:**
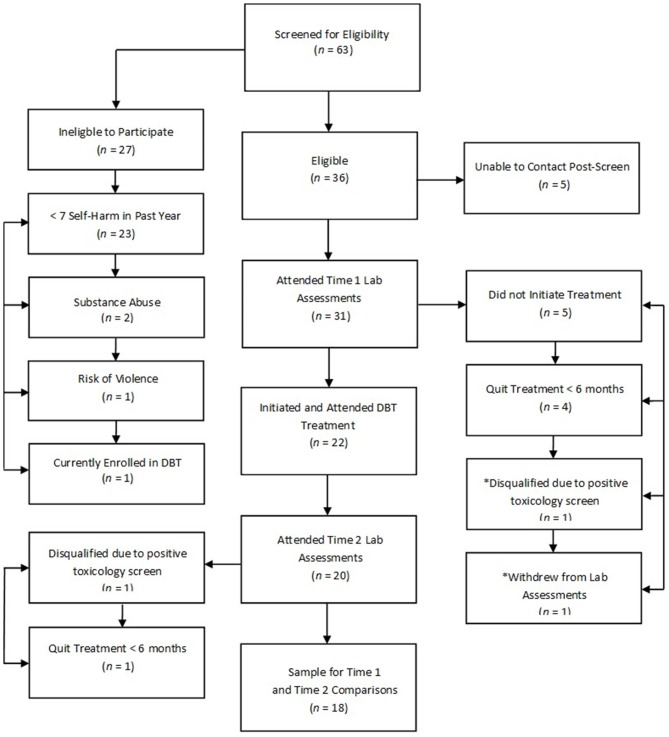
**Participant flow chart detailing completion of treatment and study procedures.** Note: An asterisk (*) indicates that the participant did not attend laboratory procedures but did complete 7 months of dialectical behavior therapy (DBT).

The current study adopted a naturalistic design by recruiting patients with BPD that were being treated as part of regular clinical services in the BPD Clinic at the Centre for Addiction and Mental Health in Toronto, Canada. Thirty-one participants with BPD reporting at least seven episodes of self-harm in the past 12 months consented and enrolled in the study. Figure 1 depicts flow of participants through the study. Individuals not eligible for the study based on a phone screen did not significantly differ from eligible individuals according to age [*t*_(52)_ = 0.89, *p* = 0.38] or gender (*p* = 0.69, two-tailed Fisher’s exact test). Participants eligible for the study were 18–65 years old, fluent in English, capable to provide written informed consent, had a current (at least past 5 years) diagnosis of BPD, and reported at least seven episodes of self-harm in the past year. Participants were excluded if they had a lifetime psychotic disorder, current substance dependence, neurological or medical illness that could impact brain function (e.g., significant head trauma, seizure disorder, or stroke), significant manual, visual or hearing impairment, or estimated IQ less than 80 on the Wechsler Test of Adult Reading (Wechsler, 2002).

### Procedure

The present study was approved by the Research Ethics Board of the Centre for Addiction and Mental Health, and the Social Sciences, Humanities and Education Research Ethics Board at the University of Toronto. After complete description of the study to the participants, written informed consent was obtained. Participants completed the Structured Clinical Interview for DSM-IV Axis I Disorders—Patient Edition (First et al., 2002), Structured Interview for DSM-IV Personality (Pfohl et al., 1995), Montgomery–Åsberg Depression Rating Scale (MADRS; Montgomery and Asberg, 1979), Young Mania Rating Scale (YMRS; Young et al., 1978), and Zanarini BPD Rating Scale (ZAN-BPD; Zanarini et al., 2003). Frequencies of self-harming behaviors were assessed with a modified Parasuicide Count (Comtois and Linehan, 1999). Diagnostic assessments were administered by Master’s and doctoral level research assistants directly supervised by a licensed psychologist (ACR). Results of diagnostic assessments were reviewed in a multidisciplinary “best estimate” diagnostic meeting (Klein et al., 1994).

Participants completed functional near-infrared spectroscopy (fNIRS) neuroimaging procedures prior to beginning DBT and after approximately 7 months of treatment (*M* = 30.20 weeks, *SD* = 13.25). A 7-month timeframe was chosen because the most substantial reductions in self-harm are observed well within this period for self-harming patients treated with DBT (McMain et al., 2009). fNIRS is a neuroimaging technique that introduces light in the near-infrared range at the level of the scalp and monitors changes in the optical properties of the local vasculature according to the differential absorption and back-scattering of photons. The fNIR Imager 1000^®^ (fNIR Devices, Potomac, MD, USA) is a continuous-wave fNIRS system that was used to deliver two wavelengths of light (730 and 850 nm) that were measured continuously at 500 ms intervals (2 Hz) in 16 channels (4 light sources and 10 photo-detectors). Source-detector separation was 2.5 cm, yielding a 1.25 cm depth of penetration. The probe (18 cm × 6 cm × 0.8 cm) was aligned with electrode positions F_7_, F_P1_, F_P2_, and F_8_ (which correspond to Brodmann areas 9, 10, 45, and 46, respectively) based on the international 10–20 electroencephalography system (Jasper, 1958). Specific details regarding placement of the probe are provided in Ayaz et al. (2006). Participants sat in a dark room in front of a computer monitor. After securing the fNIRS sensor pad on the participant’s forehead using Velcro^®^ straps, participants were asked to directly stare at a crosshair fixation for 10 s to establish baseline parameters. Participants then completed the Scarborough Non-Affective Go/No-go Task, a previously validated measure of motor inhibitory control known to activate bilateral medial and inferior frontal gyri during response inhibition (Rodrigo et al., 2014). The task was chosen for its relevance to controlling motor behaviors in participants with BPD who are actively engaging in physically self-injurious behaviors. Briefly, the task presents participants with either green (“go”) or red (“no-go”) circles at the center of a computer monitor and asks them to press a button with their right hand to “go” stimuli (90 trials) and withhold their response to “no-go” stimuli (30 trials). The inter-trial interval was jittered at increments of 500 ms (ranging from 4000–6000 ms, *M* = 5000 ms) to discourage anticipatory responding. From the raw light intensity measurements, relative concentrations of oxygenated hemoglobin (oxy-Hb) were calculated using the modified Beer-Lambert law (Cope and Delpy, 1988), which calculates the absorption of light for a given substance’s concentration. Image reconstruction was rendered using topographic tools available in fNIRSoft^®^ Professional Edition (Ayaz, 2010), which maps fNIRS activation data onto magnetic resonance imaging templates.

Participants enrolled in a standard outpatient DBT program that included both individual (1 h/week) and group psychotherapy (2 h/week; details of the treatment program are described in McMain et al., 2009). Overall, this treatment focused on eliminating behavioral dysregulation through the development of more effective coping strategies, which in turn are balanced with validation. Twenty-one participants completed treatment; however, pre-treatment neuroimaging data were unavailable for one participant that provided a positive urine toxicology screen and was ineligible to complete subsequent study procedures. Seven-month neuroimaging data were available for 18 participants who completed treatment (“treatment completers”). Neuroimaging data were unavailable at the 7-month assessment for two additional participants who continued to be enrolled in treatment because one provided a positive urine drug screen at the 7-month assessment and the second was lost to follow-up but clinic notes indicated that the participant remained in treatment. Five participants dropped out of treatment and four qualified for the study but never initiated treatment after completing pre-treatment diagnostic and neuroimaging assessments (collectively referred to as “treatment non-completers”, *n* = 9). Participants who completed treatment did not differ from non-completers in pre-treatment rate of self-harm (*z* = 1.06, *p* = 0.29; independent-samples Mann-Whitney *U* Test) or in age [*t*_(28)_ = 1.98, *p* = 0.06], gender (*p* = 0.25, two-tailed Fisher’s exact test), or IQ [*t*_(28)_ = 0.72, *p* = 0.47].

### Statistical Analysis

Neuroimaging data were analyzed using multilevel generalized linear models that nested all observations (μmol/l oxy-Hb measured at 2 Hz) within participants. The first set of analyses investigated pre-treatment motor inhibitory control -related PFC activation for all participants who completed neuroimaging. The neural activation changes that were observed for those who completed 7 months of treatment were then investigated by examining interactions between Condition (no-go vs. crosshair fixation) and Time (pre-treatment vs. 7-month assessments) in each of 16 channels. Simple effects of the significant interactions from this set of analyses were then probed (Aiken et al., 1991) to examine activation patterns prior to treatment, and separately, after 7 months of treatment. The second set of analyses examined the three-way interaction between Condition, Time and Change-in-Self-Harm-Rate. Treatment completers were compared with non-completers by exploring significant interactions between treatment completion status and inhibitory control-related neural activation in the PFC. Significant interactions were subsequently investigated to identify patterns of activation displayed by participants who showed higher symptom improvement (i.e., 1 SD above the mean) and lower symptom improvement (i.e., 1 SD below the mean) separately at each assessment time point. The final set of analyses examined differences in PFC activation between participants who completed 7 months of treatment vs. those who did not (Condition and Treatment-Completion-Status interaction), and then probing simple effects based on significant interactions. Type I error for whole-probe interaction analyses was controlled using the False-Discovery Rate approach (Benjamini and Hochberg, 1995; Benjamini et al., 2001) and *p*-values less than 0.05 were reported as statistically significant. Effect sizes (semi-partial *R*^2^) are reported where appropriate.

## Results

### Participant Characteristics

The mean age of participants was 28.65 (*SD* = 10.04) and their estimated IQ was 105.68 (*SD* = 8.20). Most participants were women (90.30%) and right-hand dominant (87.10%). The ethnic-racial composition of the sample according to 2011 Canadian census categories was as follows: Black (3.23%), Latin American (16.13%), South Asian (6.45%), White/Caucasian (64.52%) and other (9.68%). Table 1 presents frequencies of subtypes of self-harm and suicidal intent between pre-treatment and 7-month assessments. Cumulatively, rates of self-harm were significantly reduced after approximately 7 months of treatment (*z* = −3.36, *p* = 0.001; related-samples Wilcoxon Signed Rank Test). BPD symptom severity did not significantly change from pre-treatment assessments (ZAN-BPD total score *M* = 18.25, *SD* = 6.51) to 7 months of DBT (*M* = 13.70, *SD* = 5.56; *t* = 1.97, *p* = 0.06).

**Table 1:** **Rates of self-harm for patients with borderline personality disorder (*N* = 18) prior to beginning treatment and after 6 months of dialectical behavior therapy**.

	% Endorsed	Monthly Rate *M (SD)*	% of All Self-Harm	% Suicidal
**Before starting treatment**
Cutting	66.67	2.56 (7.06)	37.74	0
Overdose	27.78	0.04 (0.07)	0.54	12.5
Hanging	1.11	0.02 (0.06)	0.27	25
Asphyxiation	5.56	0.00 (0.02)	0.07	100
Burning	27.78	0.14 (0.36)	1.09	100
Jumping	5.56	0.00 (0.02)	0.07	0
Shooting	5.56	0.00 (0.02)	0.07	0
Drowning	5.56	0.01 (0.04)	0.14	100
Stabbing	16.67	0.06 (0.20)	0.82	0
Hitting	66.67	1.50 (3.46)	22.07	0
Other	55.56	2.52 (4.26)	37.13	0
**After 7 months of treatment**
Cutting	38.89	4.66 (16.92)	74.22	0
Overdose	16.67	0.06 (0.04)	0.94	0
Hanging	0	0.00 (0.00)	0	0
Asphyxiation	0	0.00 (0.00)	0	0
Burning	11.11	0.03 (0.09)	0.31	50
Jumping	0	0.00 (0.00)	0	0
Shooting	0	0.00 (0.00)	0	0
Drowning	0	0.00 (0.00)	0	0
Stabbing	11.11	0.23 (0.89)	3.59	0
Hitting	38.89	0.56 (1.23)	8.91	35.09
Other	55.56	0.75 (1.10)	12.03	1.3

*Note: % Endorsed = proportion of patients that endorsed each type of self-harm. % of All Self-Harm = proportion of a specific type of self-harm relative to the frequency of all self-harm. % Suicidal = proportion of self-harm with suicidal intent. Patients showed significant reductions in hitting (z = −2.50, p = 0.01; related- samples Wilcoxon Signed Rank Test) and other self-harm (z = −2.20, p = 0.03; related-samples Wilcoxon Signed Rank Test) after treatment*.

Psychiatric diagnostic co-morbidities for participants that qualified for the study and completed pre-treatment psychodiagnostic assessments (*n* = 29) are presented in Table 2. A majority of participants (64.71%) who completed treatment were taking medications (alone or in combination) at the time of the study: sedatives (*n* = 4), antidepressants (*n* = 8), mood stabilizers (*n* = 2), and antipsychotics (*n* = 4).

**Table 2:** **A summary of psychiatric diagnostic comorbidities within the final sample (*N* = 29)**.

Disorder	*n*	Percent in final sample
**Bipolar I disorder**	2	6.90%
**Bipolar II disorder**	1	3.45%
**Major depressive disorder**
Current	13	44.83%
Past	10	34.48%
**Dysthymic disorder**	1	3.45%
**Alcohol abuse**
Current	1	3.45%
Past	4	13.79%
**Alcohol dependence (past)**	13	44.83%
**Substance dependence (past)**	7	24.14%
Sedative	1	3.45%
Cannabis	6	20.69%
Opioid	1	3.45%
Cocaine	1	3.45%
Stimulant	1	3.45%
**Polysubstance dependence (past)**	1	3.45%
**Panic disorder**
Current	9	31.03%
Past	2	6.90%
**Agoraphobia without panic disorder (current)**	2	6.90%
**Social phobia**
Current	1	3.45%
Past	2	6.90%
**Specific phobia**
Current	1	3.45%
Past	2	6.90%
**Obsessive-compulsive disorder**
Current	6	20.69%
Past	3	10.34%
**Posttraumatic stress disorder**
Current	12	41.38%
Past	3	10.34%
**Generalized anxiety disorder**	3	10.34%
**Anorexia nervosa (past)**	4	13.79%
**Bulimia nervosa**
Current	1	3.45%
Past	4	13.79%
**Paranoid personality disorder**	3	10.34%
**Antisocial personality disorder**	5	17.24%
**Avoidant personality disorder**	8	27.59%
**Dependent personality disorder**	4	13.79%
**Obsessive-compulsive personality disorder**	3	10.34%

Participants on average reported moderate depression on the MADRS (*M* = 23.43, *SD* = 8.40) and minimal symptoms of mania on the YMRS (*M* = 9.19, *SD* = 5.78). After approximately 7 months of DBT, symptoms of depression (*M* = 19.43, *SD* = 7.90) and mania (*M* = 6.76, *SD* = 4.61) did not markedly change (*p*’s > 0.05, two-tailed repeated-measures *t*-tests). Participants reported minimal levels of suicidal ideation on the Modified Scale for Suicidal Ideation (Miller et al., 1986) before starting DBT (*M* = 3.11, *SD* = 2.61) and after 7 months of treatment (*M* = 2.61, *SD* = 3.35; *t* = 0.36, *p* = 0.73). Treatment completers did not differ from non-completers in pre-treatment levels of depression, mania, suicidal ideation, and BPD symptom severity (*p*’s ≥ 0.23; independent-samples *t*-tests).

### Behavioral Performances on the Motor Inhibitory Control Task

Accuracy on the go/no-go task (sum of true positive and true negative responses) was high for participants with BPD prior to starting DBT (*M* = 98.82%, *SD* = 1.15) and after approximately 7 months of treatment (*M* = 96.67%, *SD* = 2.61). More specifically, the number of commission errors during the no-go condition was low at pre-treatment (*M* = 2.44, *SD* = 2.25), and following approximately 7 months of treatment (*M* = 1.00, *SD* = 1.03). Participants did not differ in their accuracy (*z* = −0.12, *p* = 0.91; related-samples Wilcoxon Signed Rank Test) or response times on true positive responses (*z* = 0.65, *p* = 0.52; related-samples Wilcoxon Signed Rank Test) between the pre-treatment assessment and after 7 months of DBT. Participants, on the other hand, demonstrated a significant difference between pre- and post-treatment on commission errors (*z* = −2.22, *p* = 0.03; related-samples Wilcoxon Signed Rank Test). This finding, however, appeared to be driven by two participants who demonstrated a noticeable change in commission errors across the two time points (−23.33% change in commissions; *M* = −4.8%, *SD* = 8.57). Treatment completers did not differ from non-completers in accuracy (*z* = −0.10, *p* = 0.92; Independent-Samples Mann-Whitney *U* Test) or response times on true-positive trials (*z* = −0.86, *p* = 0.39; Independent-Samples Mann-Whitney *U* Test) on the go/no-go task. Additionally, no differences were observed between completers and non-completers with regard to commission errors during the no-go condition (*z* = −0.50, *p* = 0.62; Independent-Samples Mann-Whitney *U* Test).

### Pre-Treatment PFC Activation and Changes through Treatment

**Figure 2:**
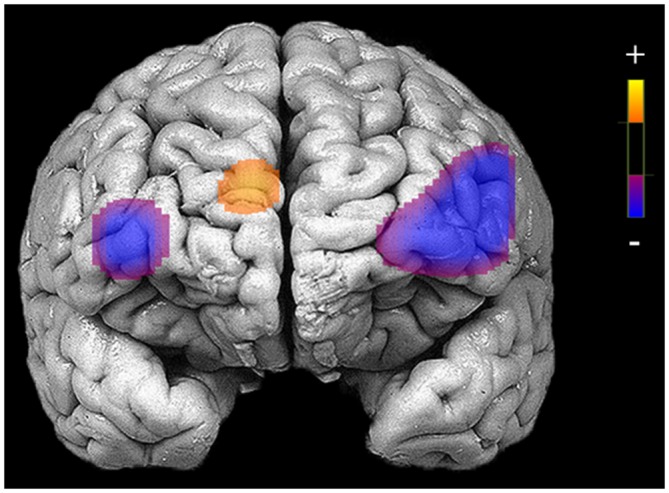
**Areas of reduced activation during response inhibition (no-go blocks minus cross-hair fixation) in bilateral medial and inferior frontal gyri and higher activation in medial prefrontal cortex (PFC) for patients who completed neuroimaging procedures at the pre-treatment assessment (*N* = 29)**.

**Figure 3:**
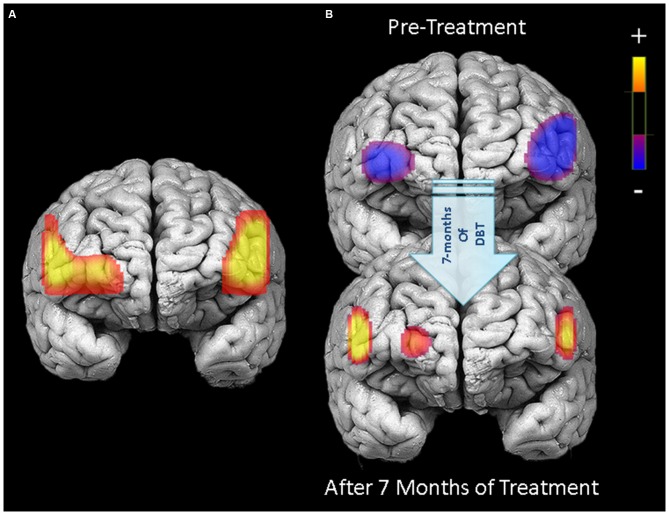
**PFC activation during response inhibition (no-go blocks minus cross-hair fixation) for patients who completed 7 months of treatment (*N* = 18).** Left panel **(A)** displays areas in the PFC showing significant treatment-related changes in activation between pre-treatment and 7-month neuroimaging assessments (channels with significant Condition × Time interactions). Right panel **(B)** illustrates PFC activation during response inhibition before starting treatment (upper) and after 7 months of DBT (lower).

Before beginning DBT, all participants with BPD who completed pre-treatment neuroimaging (*n* = 29) showed less activation in bilateral DLPFC and more activation in right medial PFC during response inhibition as compared to cross-hair fixation (*p’*s < 0.05; Table 3; Figure 2). For treatment completers (*n* = 18), participants also showed less activation in bilateral DLFPC before beginning treatment, and as shown in Figure 3, they showed treatment-related increases in activation in more widespread areas of medial aspects of the DLPFC bilaterally and right medial PFC. After 7 months of treatment, participants displayed higher levels of activation during response inhibition primarily in right DLPFC and to a lesser extent in the homologous region in the left PFC (*p*’s < 0.01). They also showed higher activation in the right medial PFC (*p* < 0.01). The main interactions for these analyses are summarized in Table 4, while the simple effects analyses are summarized in Table 5.

**Table 3:** **Multilevel analyses comparing pre-treatment levels of oxygenated hemoglobin in all 16 channels during No-Go and cross-hair fixation blocks**.

fNIRS Channel	*b*	*SE*	*df*	*t*	$R_{\beta }^{2}$
1	−0.0961	0.0133	35142.22	−7.2510**	0.0015
2	−0.0511	0.0136	37903.01	−3.7550**	0.0004
3	−0.0489	0.0127	38347.04	−3.8460**	0.0004
4	−0.0743	0.0144	37447.01	−5.1680**	0.0007
5	0.0107	0.0126	38394.07	0.8520	0.0000
6	−0.0399	0.0146	37936.03	−2.7250*	0.0002
7	0.0224	0.0129	36265.72	1.7360	0.0001
8	−0.0152	0.0160	32284.60	−0.9520	0.0000
9	0.0376	0.0147	34075.36	2.5560*	0.0002
10	0.0095	0.0148	36608.12	0.6420	0.0000
11	0.0081	0.0118	38263.18	0.6840	0.0000
12	−0.0300	0.0135	37868.06	−2.2200	0.0001
13	−0.0168	0.0121	39050.07	−1.3960	0.0000
14	−0.0838	0.0157	39148.03	−5.3480**	0.0007
15	−0.0227	0.0142	36182.99	−1.6010	0.0001
16	0.0010	0.0157	38275.14	0.0640	0.0000

Note: **p < 0.001, *p < 0.05. All models were estimated with an unstructured covariance matrix and the Satterthwaite method of estimating degrees of freedom. Significance levels were corrected using the False Discovery Rate approach.

**Table 4:** **Multilevel analyses in all 16 channels examining the interaction between Condition (no-go vs. cross-hair fixation) and Time (pre-treatment vs. 7-month assessments)**.

fNIRS Channel	*b*	*SE*	*df*	*t*	$R_{\beta }^{2}$
1	0.0671	0.0097	58357.04	6.9430**	0.0008
2	0.0615	0.0109	61952.94	5.6670**	0.0005
3	0.0282	0.0094	62573.96	2.9980*	0.0001
4	0.0551	0.0112	60260.94	4.9010**	0.0004
5	−0.0086	0.0098	62525.01	−0.8820	0.0000
6	0.0046	0.0115	62449.96	0.3980	0.0000
7	0.0012	0.0109	59390.52	0.1070	0.0000
8	0.0127	0.0127	54627.41	1.0050	0.0000
9	−0.0038	0.0116	57034.33	−0.3310	0.0000
10	−0.0106	0.0124	58427.21	−0.8600	0.0000
11	0.0204	0.0100	59973.13	2.0500	0.0001
12	0.0400	0.0110	61945.00	3.6290**	0.0002
13	0.0170	0.0099	61452.01	1.7190	0.0000
14	0.0442	0.0125	63002.99	3.5390**	0.0002
15	0.0353	0.0106	60197.88	3.3360*	0.0002
16	0.0806	0.0128	62765.93	6.2710**	0.0006

Note: **p < 0.001, *p < 0.05. All models were estimated with an unstructured covariance matrix and the Satterthwaite method of estimating degrees of freedom. Significance levels were corrected using the False Discovery Rate approach.

**Table 5:** **Multilevel analyses examining simple effects for channels with significant interactions between Condition (no-go vs. cross-hair fixation) and Time (pre-treatment vs. 7-month assessments)**.

fNIRS Channel	*b*	*SE*	*df*	*t*	$R_{\beta }^{2}$
**Pre-Treatment**
1	−0.0976	0.0124	58357.18	−7.8800**	0.0021
2	−0.0525	0.0137	61952.97	−3.8230**	0.0005
3	−0.0500	0.0119	62573.99	−4.2020**	0.0006
4	−0.0758	0.0139	60260.97	−5.4620**	0.0010
12	−0.0306	0.0139	61945.06	−2.1930*	0.0002
14	−0.0864	0.0156	63002.98	−5.5350**	0.0010
15	−0.0215	0.0136	60197.92	−1.5850	0.0001
16	0.0005	0.0162	62766.45	0.0320	0.0000
**After 7 months of treatment**	
1	0.0366	0.0148	58356.94	2.4660*	0.0002
2	0.0705	0.0168	61952.93	4.1940**	0.0006
3	0.0064	0.0146	62573.94	0.4400	0.0000
4	0.0344	0.0177	60260.92	1.9450	0.0001
12	0.0495	0.0171	61944.96	2.8940*	0.0003
14	0.0020	0.0195	63002.99	0.1040	0.0000
15	0.0492	0.0163	60197.85	3.0220*	0.0003
16	0.1617	0.0199	62765.59	8.1040**	0.0021

Note: **p < 0.001, *p < 0.05. All models were estimated with an unstructured covariance matrix and the Satterthwaite method of estimating degrees of freedom. Significance levels are FDR corrected.

### Associations between Changes in Symptom Severity and PFC Activation

**Figure 4:**
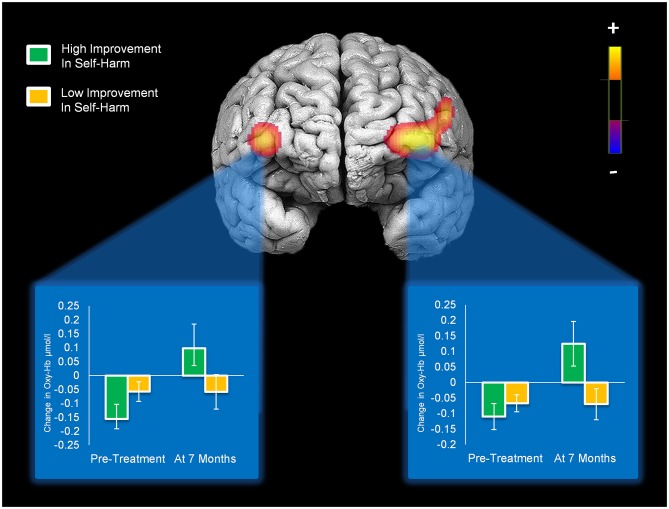
**Activation in bilateral medial/inferior frontal gyri that significantly changed with self-harm frequency after 7 months of DBT (significant Condition × Time × Change-in-Self-Harm interactions).** Bar graphs show levels of activity in each cluster of activation for patients who showed high vs. low improvements in self-harm at pre-treatment and 7-month assessments.

Reductions in self-harm were significantly associated with changes in activation in right DLPFC (Condition × Time × Change-in-Self-Harm, *p* < 0.01). Prior to beginning DBT, participants who showed the greatest reductions in self-harm displayed less activation in right DLPFC (*p* < 0.01) compared to those with the least improvements in self-harm after 7 months. Whereas participants seeing the greatest reduction in self-harm had less activation in right DLPFC before starting treatment, they demonstrated the greatest increase in activation in this region after 7 months of treatment (*p* < 0.01). Within a larger portion of the left DLPFC, a similar pattern of self-harm associations was found (*p*’s < 0.01), with the exception that participants with the greatest and least improvements did not differ in level of activation before beginning DBT (these findings are summarized in Figure 4). Statistically controlling for changes in depression, mania, and BPD symptom severity did not change the pattern of activation in bilateral PFC and resulted in a more statistically significant result (Condition × Time × Change-in-Self-Harm interactions in both regions remained significant, *p*’s < 0.01). The main interactions for these analyses are summarized in Table 6, while the simple effects analyses are summarized in Table 7.

**Table 6:** **Multilevel analyses in all 16 channels examining the interaction between Condition (no-go vs. cross-hair fixation), Time (pre-treatment vs. 7-month assessments) and Changes-In-Self-Harm**.

fNIRS Channel	*b*	*SE*	*df*	*t*	$R_{\beta }^{2}$
1	0.0024	0.0007	58350.03	3.3780**	0.0002
2	0.0016	0.0008	61945.93	1.9160	0.0001
3	−0.0010	0.0007	62566.95	−1.4170	0.0000
4	0.0042	0.0009	60253.93	4.5750**	0.0003
5	0.0003	0.0007	62517.99	0.3980	0.0000
6	0.0025	0.0009	62442.98	2.8830*	0.0001
7	0.0004	0.0008	59383.06	0.4380	0.0000
8	0.0021	0.0010	54620.06	2.1260	0.0001
9	−0.0004	0.0008	57027.22	−0.4560	0.0000
10	0.0006	0.0010	58420.01	0.6640	0.0000
11	−0.0020	0.0011	59966.06	−1.9220	0.0001
12	0.0011	0.0008	61937.97	1.3010	0.0000
13	0.0004	0.0011	61444.97	0.3690	0.0000
14	0.0034	0.0009	62995.97	3.6410**	0.0002
15	0.0015	0.0008	60190.88	1.9440	0.0001
16	0.0019	0.0010	62758.65	1.9740	0.0001

Note: **p < 0.001, *p < 0.05. All models were estimated with an unstructured covariance matrix and the Satterthwaite method of estimating degrees of freedom. Significance levels were corrected using the False Discovery Rate approach.

**Table 7:** **Multilevel analyses examining the simple effects for channels with significant Condition (no-go vs. cross-hair fixation) × Time (pre-treatment vs. 7-month assessments) × Changes-In-Self-Harm interactions**.

		fNIRS Channel	*b*	*SE*	*df*	*t*	$R_{\beta }^{2}$
**Pre-Treatmet**
Low Treatment Response	Left DLPFC	1	−0.0802	0.0137	58350.20	−5.8390**	0.0006
		4	−0.0578	0.0157	60253.96	−3.6890**	0.0002
		6	−0.0415	0.0162	62442.97	−2.5630*	0.0001
	Right DLPFC	14	−0.0575	0.0175	62995.97	−3.2900**	0.0002
High Treatment Response	Left DLPFC	1	−0.1404	0.0209	58350.43	−6.7170**	0.0008
		4	−0.1208	0.0231	60253.96	−5.2330**	0.0005
		6	−0.0321	0.0249	62443.13	−1.2920	0.0000
	Right DLPFC	14	−0.1560	0.0266	62996.05	−5.8600**	0.0005
**After 7 months of DBT**
Low Treatment Response	Left DLPFC	1	−0.0800	0.0233	58349.95	−0.3430	0.0000
		4	−0.0617	0.0300	60253.93	−2.0560*	0.0001
		6	−0.1067	0.0284	62442.95	−3.7570**	0.0002
	Right DLPFC	14	−0.0580	0.0310	62995.98	−1.8710	0.0001
High Treatment Response	Left DLPFC	1	0.1092	0.0329	58349.95	3.3230**	0.0002
		4	0.1905	0.0433	60253.93	4.4040**	0.0003
		6	0.0882	0.0401	62442.95	2.1990*	0.0001
	Right DLPFC	14	0.0977	0.0436	62995.96	2.2410*	0.0001

Note: **p < 0.01, *p < 0.05. All models were estimated with an unstructured covariance matrix and the Satterthwaite method of estimating degrees of freedom. Significance levels are FDR corrected.

### Pre-Treatment Activation Differences Between Completers and Non-Completers

**Figure 5:**
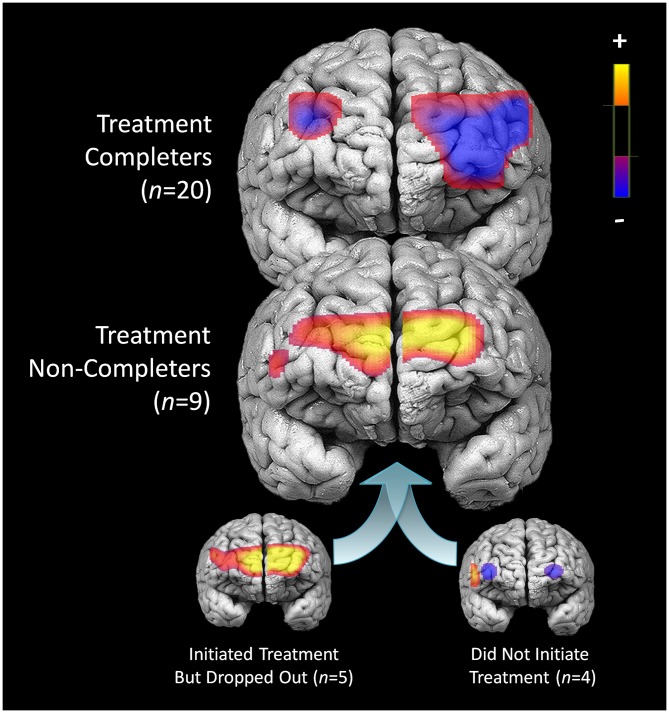
**Pre-treatment differences in activation during response inhibition for patients who completed treatment and did not complete treatment**.

Compared to participants that either dropped out or did not initiate treatment (*n* = 9), participants that completed 7 months of treatment (*n* = 20) showed less pre-treatment activation during response inhibition in a large cluster within the left DLPFC (Figure 5). Additionally, they showed less activation in a smaller region within the right DLPFC. Conversely, non-completers displayed greater activation mainly in the medial PFC/frontal pole and right inferior frontal gyrus prior to beginning DBT. This pattern of higher activation was found in a larger spatial extent within the medial PFC and right DLPFC in participants who dropped out (*n* = 5) as compared to those who completed approximately 7-months of treatment (*p*’s < 0.05). Participants who did not initiate treatment, on the other hand, showed less activation in two smaller areas of the PFC bilaterally and showed higher activation to a similar spatial extent in the right lateral PFC, as compared to those who initiated and remained in treatment (*p*’s < 0.05). The main interactions for these analyses are summarized in Table 8, while the simple effects analyses are summarized in Tables 9–11.

**Table 8:** **Multilevel analyses in all 16 channels examining the interaction between Condition (no-go vs. cross-hair fixation) and Treatment Completion Status (completers vs. non-completers)**.

fNIRS Channel	*b*	*SE*	*df*	*t*	$R_{\beta }^{2}$
1	−0.0480	0.0145	35139.24	−3.3030**	0.0003
2	−0.0123	0.0155	37900.06	−0.7960	0.0000
3	−0.0540	0.0142	38344.06	−3.8020**	0.0004
4	−0.0414	0.0162	37444.04	−2.5600*	0.0002
5	−0.1156	0.0134	38391.11	−8.6120**	0.0019
6	−0.0704	0.0159	37933.02	−4.4130**	0.0005
7	−0.1047	0.0141	36263.39	−7.4220**	0.0015
8	−0.0298	0.0174	32282.18	−1.7110	0.0001
9	−0.0876	0.0160	34072.27	−5.4620**	0.0009
10	−0.0634	0.0163	36605.21	−3.8980**	0.0004
11	−0.0634	0.0127	38260.13	−4.9810**	0.0006
12	0.0213	0.0147	37865.12	1.4460	0.0001
13	−0.0581	0.0131	39047.12	−4.4220**	0.0005
14	−0.0337	0.0169	39145.02	−1.9940	0.0001
15	−0.0050	0.0151	36179.00	−0.3300	0.0000
16	−0.0622	0.0176	38272.81	−3.5380**	0.0003

Note: **p < 0.001, *p < 0.05. All models were estimated with an unstructured covariance matrix and the Satterthwaite method of estimating degrees of freedom. Significance levels were corrected using the False Discovery Rate approach.

**Table 9:** **Multilevel analyses examining the simple effects for significant interaction between Condition (no-go vs. cross-hair fixation) and Treatment Completion Status (completers vs. non-completers)**.

fNIRS Channel	*b*	*SE*	*df*	*t*	$R_{\beta }^{2}$
**Completers (*n* = 19)**
1	−0.1243	0.0158	35139.2230	−7.8820**	0.0018
3	−0.0788	0.0149	38344.0410	−5.2760**	0.0007
4	−0.0966	0.0168	37443.9980	−5.7470**	0.0009
5	−0.0632	0.0152	38391.0470	−4.1510**	0.0004
6	−0.0822	0.0175	37933.0210	−4.7050**	0.0006
7	−0.0390	0.0153	36262.2080	−2.5430*	0.0002
9	−0.0148	0.0176	34072.3360	−0.8450	0.0000
10	−0.0268	0.0174	36605.0160	−1.5360	0.0001
11	−0.0316	0.0142	38260.2510	−2.2200*	0.0001
13	−0.0517	0.0144	39047.0440	−3.5870*	0.0003
16	−0.0324	0.0183	38271.6950	−1.7690	0.0001
**Non-Completers (*n* = 10)**
1	−0.0283	0.0244	35139.2390	−1.1620	0.0000
3	0.0291	0.0241	38344.0710	1.2060	0.0000
4	−0.0139	0.0276	37444.0590	−0.5030	0.0000
5	0.1680	0.0221	38391.1350	7.5970**	0.0015
6	0.0585	0.0267	37933.0140	2.1940*	0.0001
7	0.1705	0.0237	36263.8770	7.1950**	0.0014
9	0.1604	0.0269	34072.2410	5.9750**	0.0010
10	0.1001	0.0275	36605.2920	3.6420**	0.0004
11	0.0951	0.0211	38260.0710	4.5110**	0.0005
13	0.0645	0.0220	39047.1570	2.9360**	0.0002
16	0.0921	0.0300	38273.2120	3.0660**	0.0002

Note: **p < 0.001, *p < 0.05. All models were estimated with an unstructured covariance matrix and the Satterthwaite method of estimating degrees of freedom. Significance levels are FDR corrected.

**Table 10:** **Multilevel analyses exploring simple effects of the significant Inhibitory Control × Treatment Completion interaction for those who dropped out of treatment**.

fNIRS Channel	*b*	*SE*	*df*	*t*	$R_{\beta }^{2}$
**At Pre-Treatment**
1	−0.0976	0.0124	58357.19	−7.8800**	0.0008
2	−0.0525	0.0137	61952.97	−3.8230**	0.0005
3	−0.0500	0.0119	62573.99	−4.2020**	0.0001
4	−0.0758	0.0139	60260.97	−5.4620**	0.0004
12	−0.0306	0.0139	61945.06	−2.1930*	0.0002
14	−0.0864	0.0156	63002.98	−5.5350**	0.0002
15	−0.0215	0.0136	60197.92	−1.5850	0.0002
16	0.0005	0.0162	62766.45	0.0320	0.0006
**After 7 months of DBT**
1	0.0366	0.0148	58356.94	2.4660*	0.0001
2	0.0705	0.0168	61952.93	4.1940**	0.0003
3	0.0064	0.0146	62573.94	0.4400	0.0000
4	0.0344	0.0177	60260.92	1.9450	0.0001
12	0.0495	0.0171	61944.96	2.8940**	0.0001
14	0.0020	0.0195	63002.99	0.1040	0.0000
15	0.0492	0.0163	60197.85	3.0220**	0.0002
16	0.1617	0.0199	62765.59	8.1040**	0.0010

Note: **p < 0.001, *p < 0.05. All models were estimated with an unstructured covariance matrix and the Satterthwaite method of estimating degrees of freedom. Significance levels are FDR corrected.

**Table 11:** **Multilevel analyses exploring simple effects of the significant Inhibitory Control × Treatment Completion interaction for those who never initiated treatment**.

fNIRS Channel	*b*	*SE*	*df*	*t*	$R_{\beta }^{2}$
6	−0.0338	0.0122	53960.95	−2.771*	0.0001
8	0.0140	0.0135	46651.03	1.0420	0.0000
10	0.0029	0.0123	49939.09	0.2330	0.0000
12	0.0187	0.0119	53387.97	1.5780	0.0000
14	−0.0489	0.0132	54493.98	−3.7170**	0.0003
15	0.0135	0.0111	51818.83	1.2150	0.0000
16	0.0656	0.0130	54461.77	5.0310**	0.0005

Note: **p < 0.001, *p < 0.05. All models were estimated with an unstructured covariance matrix and the Satterthwaite method of estimating degrees of freedom. Significance levels are FDR corrected.

Changes in activation for intention-to-treat analyses were examined wherein pre-treatment observations were carried forward for those who did not complete 7 months of treatment. The pattern of activation across the PFC for intention-to-treat analyses was similar to that observed in analyses using completers alone, but with smaller clusters of significant change (*p*’s < 0.5).

## Discussion

Consistent with randomized-controlled trials of DBT (Linehan et al., 2006; McMain et al., 2009), participants with BPD in the current study showed substantial declines in their frequency of self-harm over the treatment period. Prior to beginning treatment, patients had less activation in bilateral middle/inferior frontal gyri during response inhibition. After 7 months of treatment, they showed significant gains in activity in these same regions. Reductions in self-harm over the treatment period were specifically related to right DLPFC activation, even after statistically controlling for changes in depression, mania, and BPD symptom severity, and patients showing the greatest treatment gains showed lower activation in this region prior to beginning DBT. Additionally, treatment completers showed less activation in left DLPFC before starting treatment, whereas non-completers displayed higher activation in medial PFC and right inferior frontal gyrus.

Reduced activation in bilateral middle/inferior frontal gyri during response inhibition for patients with BPD prior to beginning treatment likely reflects a diminished recruitment of inhibitory control processes subserved by the PFC, especially within the left DLPFC. This pattern of activity contrasts with healthy adults on the same go/no-go task, who have shown higher levels of activity in bilateral DLPFC during response inhibition (compared to cross-hair fixation; Rodrigo et al., 2014). These results also parallel studies that have used different response inhibition tasks (e.g., emotional Stroop) in which patients with BPD show differences in neural activation in left inferior frontal gyrus under conditions of response inhibition (Wingenfeld et al., 2009; Winter et al., 2015). Overall, these findings indicate that before beginning treatment, patients with BPD characterized by marked behavioral impulsivity (i.e., active self-harm) show reduced activation in lateral areas of the PFC responsible for motor inhibitory control (Aron et al., 2004).

Despite showing significantly lower activity in bilateral medial and inferior frontal gyri prior to beginning treatment, patients exhibited increases in activity within these regions after approximately 7 months of DBT. The right lateral PFC, an area highly linked to impulse control (Aron et al., 2004), showed a specific association with changes in frequency of self-harm over the treatment period. Increases in activation within this region were related to corresponding reductions in self-harm after DBT. Remarkably, patients who went on to display the greatest improvements in self-harm exhibited the lowest activation in right DLPFC prior to beginning treatment; however, they also demonstrated the greatest increases in activation in this area after 7 months of DBT. These results suggest that self-harming patients with BPD who show the lowest pre-treatment engagement of prefrontal regions involved in motor control (as well as other aspects of self-regulation) may have the most to gain from a psychological treatment intended to increase behavioral and emotional control. Moreover, these potential neural markers of treatment response appear to be detectable even before patients begin treatment.

Patients who ultimately did not complete treatment displayed more activation in the medial PFC and right inferior frontal gyrus before starting DBT. Conversely, treatment completers showed less activation in a large region of the left DLPFC prior to beginning DBT. Heightened activity in the right inferior frontal gyrus, a region critical for impulse control, appears to represent a risk factor that may prospectively predict which patients will not complete treatment. It is possible that control processes subserved by the PFC are more efficiently recruited in this subset of patients as compared to treatment completers, who showed less activation in a roughly homologous region in the left hemisphere. Higher levels of activation in the medial PFC, a neural region involved in self-referential thinking (Gusnard et al., 2001), could also reflect an increased focus on the self in relation to others among patients at risk for treatment non-completion. Indeed, increased activation in medial PFC is observed in patients with BPD engaged in social-cognitive activities (Ruocco et al., 2010; Domsalla et al., 2014), possibly reflecting so-called “hyper-mentalizing” (i.e., heightened attention to one’s own intentional mental states and that of others), which is considered central to developmental theories of the disorder (Fonagy and Bateman, 2008). This is a speculative interpretation of these results; however, they may be relevant to social and interpersonal dynamics that could impact the degree of engagement in psychotherapy.

A main limitation of this study is its focus on the PFC, which although strongly linked to response inhibition (Rodrigo et al., 2014), is one hub of a larger brain network subserving this cognitive ability (Swick et al., 2011). Whereas the fNIRS system employed in this study primarily accesses the anterior portion of the frontal cortex, an important advantage of the technique is that it affords high temporal resolution and is both portable and cost-effective, which may facilitate its translation to clinical settings (Irani et al., 2007). For these results to have an applied clinical value, however, replication in a larger cohort of patients using a randomized-controlled design in a formal clinical trial is necessary. Indeed, prospective investigation of the predictive utility of these biomarkers will assist in validating the current findings, especially in relation to their incremental validity over other clinical measures. Furthermore, examination of the test-retest reliability of fNIRS requires further study to establish the potential clinical utility of these findings. While the present study was naturalistic in its design and included patients with BPD who were treated as part of regular clinical services and were mostly on medications and had high levels of psychiatric diagnostic comorbidity, these results may be more generalizable to the typical DBT treatment setting with self-harming patients with BPD. Additionally, factors such as age, gender, and handedness could potentially impact the results of this work. Future research with adequately large sample sizes should address these questions while also extending the present findings to interactions between response inhibition and affective processes. It is also important to note that, given the preliminary nature of the present study, it is premature to draw specific conclusions about the effectiveness of fNIRS for predicting treatment outcomes at the level of an individual patient. These results provide initial evidence for potential neuroimaging-based biomarkers that may be used to prospectively predict treatment outcomes. Importantly, these findings must be replicated in independent samples using appropriate statistical techniques to assess the sensitivity and specificity of the biomarkers for predicting treatment outcomes.

In summary, the potential implication of this research is that distinct patterns of neural activity in areas of the PFC reflecting treatment responses to DBT and attrition from therapy may be detectable even before patients start treatment. Research to validate these potential biomarkers reflecting future treatment outcomes may assist in identifying at-risk patients and intervening earlier in the course of self-harm to prevent potential serious injury and suicide. Ultimately, this work may also help to reduce self-harm in more vulnerable patients identified using these candidate biomarkers by increasing the intensity of DBT or diverting these patients toward alternative interventions.

## Author Contributions

ACR made a substantial contribution to the study conception and design, the acquisition of data, and analysis and interpretation of the data. AHR made a substantial contribution to the collection of data and analysis and interpretation of the data. SFM made a substantial contribution to the study conception and design and interpretation of the data. EP-G made a substantial contribution to the analysis and interpretation of the data. HA made a substantial contribution to the study conception and design, and analysis and interpretation of the data. PSL made a substantial contribution to the study conception and design and interpretation of the data. All listed authors made a substantial contribution to drafting the article and gave final approval of the version of the article to be published.

## Funding

This research was supported by Grant YIG-1-034-11 awarded to ACR and PSL from the American Foundation for Suicide Prevention. The content is solely the responsibility of the authors and does not necessarily represent the official views of the American Foundation for Suicide Prevention. This research was also supported in part by a New Investigator Salary Award (MSH–130177) from the Canadian Institutes of Health Research and an Early Researcher Award (ER14-10-185) from the Ministry of Research and Innovation, Province of Ontario to ACR.

## Conflict of Interest Statement

Dr. Ayaz reports that he was involved in the development of the optical brain imaging instrumentation utilized in the present research and has a minor share in the firm fNIR Devices, L.L.C., which manufactured the fNIRS device. The authors declare that the research was conducted in the absence of any commercial or financial relationships that could be construed as a potential conflict of interest.
